# Unsupervised feature learning and optimised clustering with fine-tuning for automatic semiconductor wafer defect recognition

**DOI:** 10.1038/s41598-026-63373-7

**Published:** 2026-07-23

**Authors:** M. M. Manjurul Islam, Girijesh Prasad

**Affiliations:** Intelligent Systems Research Centre, School of Computing, Engineering and Intelligent Systems, Ulster University, Londonderry, BT48 7JL U.K.

**Keywords:** Engineering, Mathematics and computing

## Abstract

Automated identification of wafer defect maps (WDMs) is critical for yield enhancement and quality control in semiconductor manufacturing. However, as defect patterns become increasingly diverse and dynamic, conventional supervised methods remain constrained by their dependence on fully labelled data and their limited adaptability to changing defect distributions. This study proposes a two-stage framework for automatic wafer defect map (WDM) recognition using predominantly unlabelled data with limited prior knowledge. First, a deep autoencoder-based representation learning approach is developed to automate feature extraction, followed by Elbow-guided K-means clustering to determine the number of clusters and capture the underlying defect structure. This stage provides an initial basis for automatic labelling without requiring exhaustive manual annotation. In the second stage, because these initial labels may become less reliable under complex data distributions and evolving defect patterns, XGBoost-based test-time training and semi-supervised refinement (TTT-SSR) is used to refine predictions by exploiting limited knowledge from the training phase. Experimental results on a benchmark mixed-WDM dataset show that the proposed framework achieves 95% classification accuracy, together with competitive precision, recall and F1-score relative to state-of-the-art methods, while also improving clustering quality across key evaluation metrics.

## Introduction

Today, semiconductors are extensively used in various fields, including consumer electronics, communication systems, smart grids, and data storage systems^1^. As the key raw material in semiconductor manufacturing, wafers play a crucial role in determining the quality of the final semiconductor products. During wafer fabrication, wafer map defects can originate from various operational and process-related factors, including process drift, equipment variation, tool variation, contamination, lithography overlay errors, etching-induced damage, deposition non-uniformity, and handling damage^2–4^. In the quest for precision and efficiency in semiconductor manufacturing, the detection and management of wafer defects are crucial for maintaining product quality and yield^5,6^. Early identification of these defects is vital to prevent defective products and optimise manufacturing^7^.

Traditionally, defect detection has relied on manual inspection or supervised learning techniques that require extensive labelled datasets for model training^8^. While effective in controlled settings, supervised learning models face significant challenges in complex manufacturing environments^9,10^. As production scales and processes evolve, new defect types inevitably emerge that are absent from historical data, thereby requiring frequent retraining for reliable performance. Furthermore, labelled data are typically scarce in real industrial settings, and the annotation of new and evolving defects is both time-consuming and costly^11^.

Several researchers have made contributions to improving supervised learning techniques for wafer defect detection^12^. For example, Adly et al.^13^ developed a randomised general regression network that combined multiple machine learning techniques for defect pattern identification in semiconductor wafer defect maps (WDMs). Tello et al.^14^ extended this work by proposing a deep-structured machine learning model that improves performance on mixed-pattern defects and addresses more complex defect scenarios in semiconductor fabrication. Similarly, Yu and Lu^15^ explored manifold learning combined with supervised techniques to improve wafer defect identification through joint local and nonlocal linear discriminant analysis. Wang et al.^16^ proposed a deformable convolutional network (DC-Net) for mixed-type WDM classification. Furthermore, Schrunner et al.^9^ compared various supervised approaches and highlighted both their effectiveness and limited adaptability. More recently, Ni et al. proposed a supervised few-shot incremental learning framework for detecting unknown wafer defects with limited samples^17^. It learns new labelled defect classes through incremental updating, but remains based on supervised class expansion rather than unsupervised structure discovery or clustering-based refinement. Overall, supervised WDM recognition methods perform well in controlled settings but require substantial labelled data and typically require retraining to accommodate new or evolving defect types^11^.

To reduce label dependence, semi-supervised and self-supervised strategies have been explored. For instance, Saqlain et al.^18^ developed a voting ensemble classifier for defect pattern identification and demonstrated the advantages of ensemble methods in improving classification accuracy. Geng et al.^19^ combined few-shot learning with self-supervised learning to enhance the classification of wafer failure patterns and demonstrated the potential of hybrid approaches that incorporate both labelled and unlabelled data.

Other semi-supervised approaches have explored evolutionary models to better handle imbalanced datasets. More recently, SWaCo^6^ introduced a self-supervised contrastive learning framework that pre-trains on large collections of unlabelled wafer bin maps and then fine-tunes a classifier on a small labelled subset to mitigate class-distribution mismatch. Other related efforts addressed data imbalance using evolutionary models such as evolutionary fuzzy ARTMAP neural networks^20^.

Unsupervised learning has gained increasing attention because it can operate without labelled data, which is attractive in manufacturing environments where defect patterns evolve and manual labelling becomes a bottleneck^21^. Li et al.^21^ demonstrated unsupervised clustering for wafer defect recognition, although the resulting number of clusters can be unstable. Hybrid systems have also been investigated. Rundo et al.^22^ combined CNN-based recognition with density-based clustering to support the discovery of emerging defect patterns, and Taha et al.^23^ introduced *DDPFinder* tools to identify dominant defect groups. Recently, Shin et al. proposed a hybrid wafer bin map framework that combines unknown-pattern detection, supervised classification, clustering, and active learning for model updating^24^. Although this approach addresses unseen defect patterns, it still lacks a unified framework that combines representation learning and refinement for automatic defect classification.

Recently, representation learning further improves clustering and recognition by providing compact feature spaces. Yu^25^ proposed a stacked denoising autoencoder to learn discriminative features from wafer maps. Other studies have shown that task-specific latent representations can improve clustering quality, confidence analysis, and interpretability in wafer map analysis^26^. An unsupervised wafer-map analysis pipeline has been proposed that combines convolutional autoencoder-based representation learning with improved deep embedded clustering (IDEC) and optional PCA refinement^27^. The results show that this unsupervised representation learning framework produces compact latent representations for high-confidence clustering and outperforms pretrained models such as DINOv2 and TIMM-ResNet on wafer-map clustering tasks. Furthermore, authors also explored generative and diffusion-based approaches for wafer map classification, which effectively mitigate class imbalance and improve robustness to rare defect patterns^28^. Despite these advances, most existing methods still focus either on clustering quality or on classification under fixed label settings.

Therefore, this paper proposes an automated wafer defect map (WDM) recognition framework that combines unsupervised representation learning, Elbow-guided optimised K-means clustering, and test-time semi-supervised refinement (TTT-SSR). In the training stage, deep autoencoder-based representation learning is used to automatically extract discriminative features from unlabelled WDM data. The resulting latent representations are then analysed using Elbow-guided K-means clustering to determine the number of clusters automatically. Furthermore, the within-cluster sum of squares (WCSS) objective function with K-means is used to assess cluster quality and identify the underlying defect structure. This phase provides the basis for the initial automatic labelling of unseen data. However, the reliability of these initial labels may decline under substantial complexity and variability in unseen samples. To address this limitation, in the testing stage we propose an automatic WDM classification process in which the initial labelling of unseen WDMs is further refined using XGBoost-based TTT-SSR^29^ with limited knowledge to improve classification performance under complex and variable unseen conditions. The key contributions of this study are summarised as follows:

- We develop an autoencoder-based latent representation learning approach for high-dimensional wafer defect maps that yields a compact and discriminative feature space for more effective clustering and classification.
- We improve the traditional K-means method by employing Elbow-guided WCSS analysis to automatically determine the number of clusters.
- One objective of the optimised clustering analysis was to enable automatic labelling of data. However, the reliability of these initial labels may decline under substantial complexity and variability in unseen samples. To address this limitation, we propose an automatic WDM classification method in which a small labelled subset is used to map clusters to defect classes, unseen samples are assigned to the nearest cluster centroid, and classification performance is further improved using XGBoost-based TTT-SSR.
- The proposed framework is validated on a benchmark dataset of mixed WDMs, demonstrating strong clustering quality and achieving a state-of-the-art classification accuracy of 95%. The method outperforms existing approaches across all major evaluation metrics.

The remainder of this paper is organised as follows. Section “Methodology” describes the proposed methodology, including the deep autoencoder-based feature extraction, the Elbow-based optimised K-means clustering algorithm, and the automatic labelling mechanism for WDMs classification. Section "Results and discussion" presents the experimental setup and results, analysing both clustering quality and classification performance. Finally, Section “Conclusion” concludes the paper and discusses potential directions for future research.

## Methodology

**Fig. 1:**
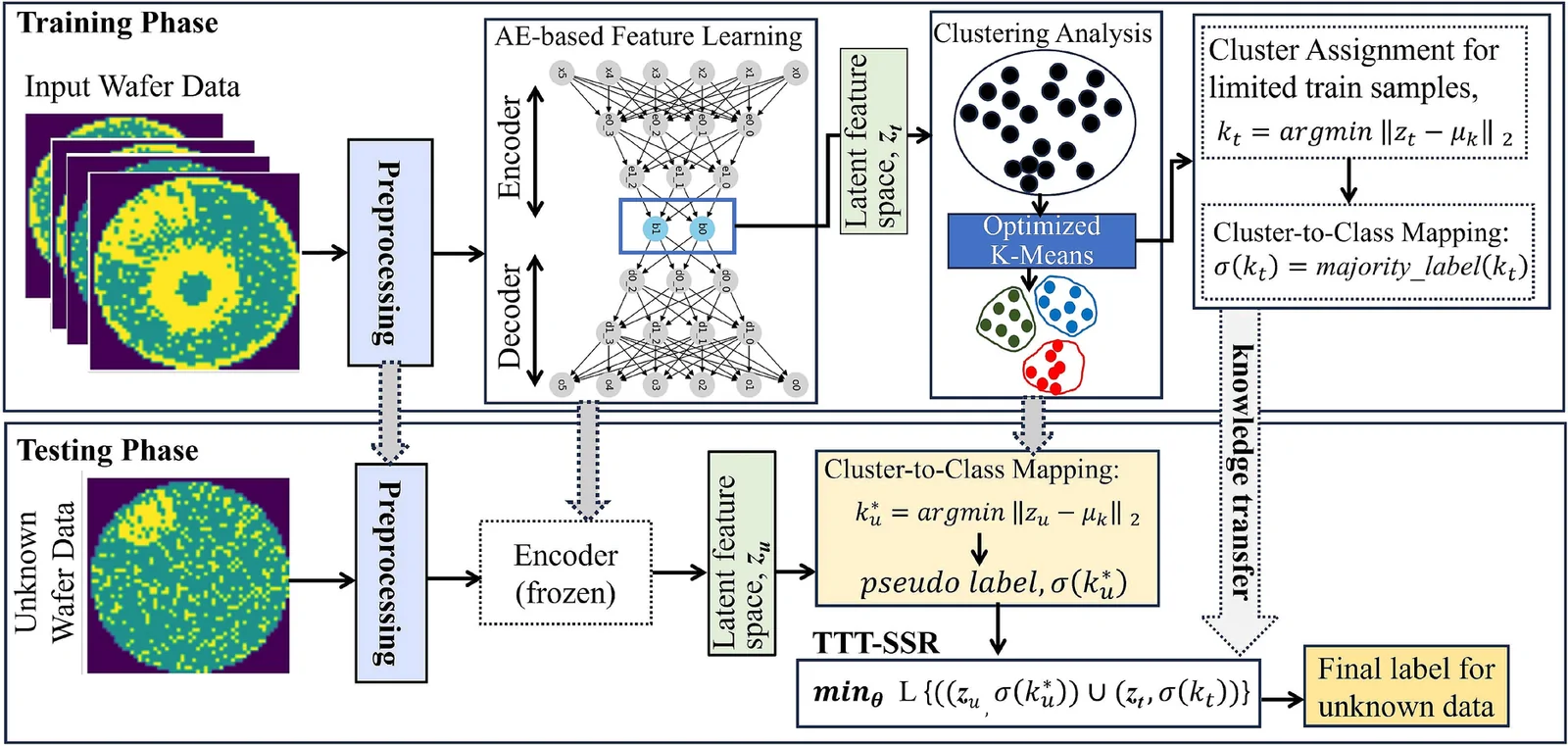
A two-stage framework of the proposed method for automatic wafer defect recognition. In the training stage, wafer defect maps are preprocessed, encoded into a latent feature space, and clustered by Elbow-guided optimised K-means to learn the underlying defect structure. In the test stage, unseen wafer defect maps are preprocessed, assigned to clusters through a nearest-centroid method, mapped to class labels through Cluster-to-Class Mapping, and refined using TTT-SSR to obtain the final prediction. Dashed arrows indicate model transfer. $\mu _k$, centroid of cluster *k*; $k_u^{*}$, index of the nearest centroid; *σ (k)*, cluster-to-class mapping; TTT-SSR, test-time training based semi-supervised refinement.

This section provides a detailed description of the proposed framework for WDMs recognition, summarised in Fig. 1. The training phase focuses on unsupervised representation learning to automate feature extraction and clustering in the latent space, whereas the testing phase uses cluster-to-class labelling and TTT-SSR to perform automatic WDM classification and improve classification performance.

### Deep autoencoder-based feature learning

We employ an autoencoder to reduce the high dimensionality of WDMs data while preserving critical features. This enables more effective clustering by extracting compact and meaningful representations from complex patterns. Autoencoding involves three steps: compressing the input to a lower-dimensional feature space via the encoder, capturing essential features in the bottleneck layer, and reconstructing the input through the decoder while minimising reconstruction error. The encoder transforms the input $x \in \mathbb {R}^{N}$, a flattened image vector, into a compressed latent representation using a series of dense layers, each performing a linear transformation followed by a nonlinear activation:

1 $$\begin{aligned} h_{i+1} = \phi (W_i h_i + b_i), \end{aligned}$$

where $h_0 = x$, $W_i$ and $b_i$ are the weights and biases, and *ϕ* is typically a ReLU activation.

The bottleneck layer captures the most salient features using fewer neurons:

2 $$\begin{aligned} z = \phi (W_z h_z + b_z), \end{aligned}$$

where $h_z$ is the final encoder output.

The decoder reconstructs the input from *z* through dense layers that expand dimensionality:

3 $$\begin{aligned} h_{j+1} = \phi (W_j h_j + b_j), \end{aligned}$$

starting with $h_0 = z$. The final layer uses a sigmoid activation to produce the output $\hat{x}$:

4 $$\begin{aligned} \hat{x} = \sigma (W_f h_f + b_f), \end{aligned}$$

where $h_f$ is the last decoder output.

The model minimises reconstruction error using mean squared error:

5 $$\begin{aligned} L(x, \hat{x}) = \frac{1}{N} \sum _{i=1}^{N} (x_i - \hat{x}_i)^2, \end{aligned}$$

optimised with Adam over multiple training epochs.

This autoencoder-based method learns compact representations that capture the critical characteristics of semiconductor wafer images. The overall training procedure is outlined in Algorithm 1, where the autoencoder is optimised to extract meaningful features by compressing and reconstructing the input data. The corresponding training settings and model configuration are summarised in Table 1 and Table 2, respectively.

**Algorithm 1:**
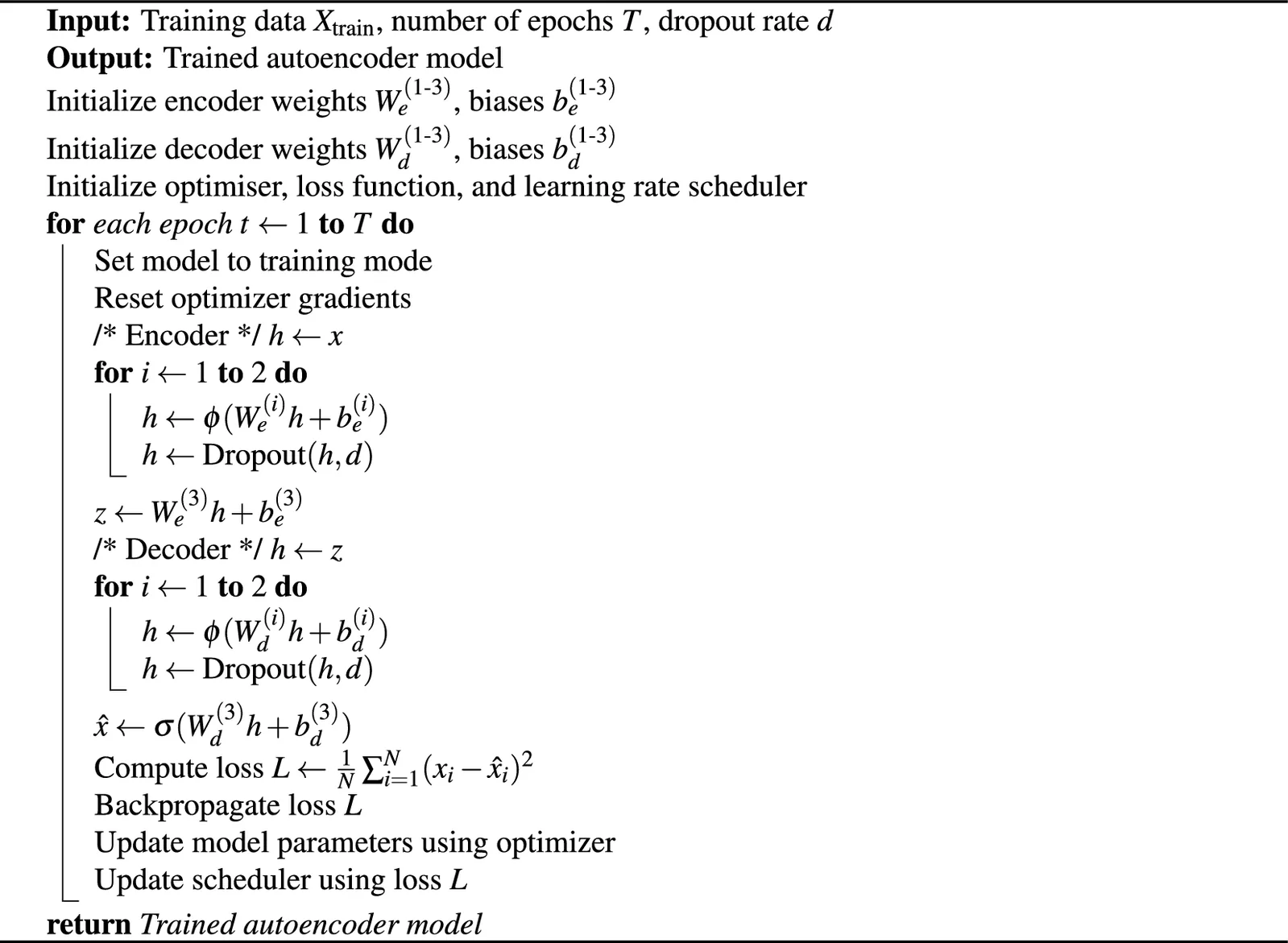
Deep autoencoder method for feature learning

**Table 1:** Experimentally selected training parameters of proposed autoencoder.

**Parameter**	**Value**
Optimizer	AdamW
Loss Function	Mean Squared Error (MSE)
Learning Rate	0.001
Epochs	50
Batch Size	Full batch
Dropout Rate	0.3
Scheduler	ReduceLROnPlateau

**Table 2:** Optimised autoencoder network architecture and hyperparameters.

**Layer Type**	**Output Dimension**	**Activation**	**Other Parameters**
Dense	256	ReLU	-
Dropout	-	-	Rate = 0.3
Dense	128	ReLU	-
Dropout	-	-	Rate = 0.3
Dense	38	-	Encoded Representation
Dense	128	ReLU	-
Dropout	-	-	Rate = 0.3
Dense	256	ReLU	-
Dropout	-	-	Rate = 0.3
Dense	2704	Sigmoid	Output Layer

### Optimised K-means for clustering

Once a compact set of features has been learned by the autoencoder, it is fed into an Elbow-based K-means clustering algorithm to determine the number of clusters automatically. The classic K-Means clustering method used in semiconductor manufacturing to group data into *K* clusters based on centroid proximity^30^. However, to adaptively identify evolving defect patterns, we integrate the Elbow method to determine the optimal *K* by minimising an objective function for compact and well-separated clusters.

#### Elbow method for optimal cluster determination

The Elbow method is a heuristic approach for selecting the optimal number of clusters in the K-Means algorithm. However, the quality of the solution depends on a precisely defined objective function, WCSS. We compute the WCSS over a range of cluster counts ($min_k$ to $max_k$), where WCSS is the sum of squared distances between data points and their nearest cluster centroids, formally defined as:

6 $$\begin{aligned} WCSS = \sum _{k=1}^K \sum _{x \in C_k} ||x - \mu _k||^2 \end{aligned}$$

where, *K* denotes the number of clusters, $C_k$ is a set of points belonging to cluster *k*, *x* represents a data point within cluster $C_k$, $\mu _k$ is the centroid of cluster $C_k$, and $||x - \mu _k||$ represents the Euclidean distance between point *x* and centroid $\mu _k$.

This measure reflects how well the data are clustered, with lower WCSS values indicating more compact and coherent clusters. The Elbow method selects the optimal number of clusters by locating the point on the WCSS curve where the rate of decrease changes noticeably, beyond which additional clusters provide little improvement. The K-means clustering procedure and the Elbow-based optimum cluster number selection used in this paper are summarised in Algorithm 2 and Algorithm 3, respectively.

**Algorithm 2:**
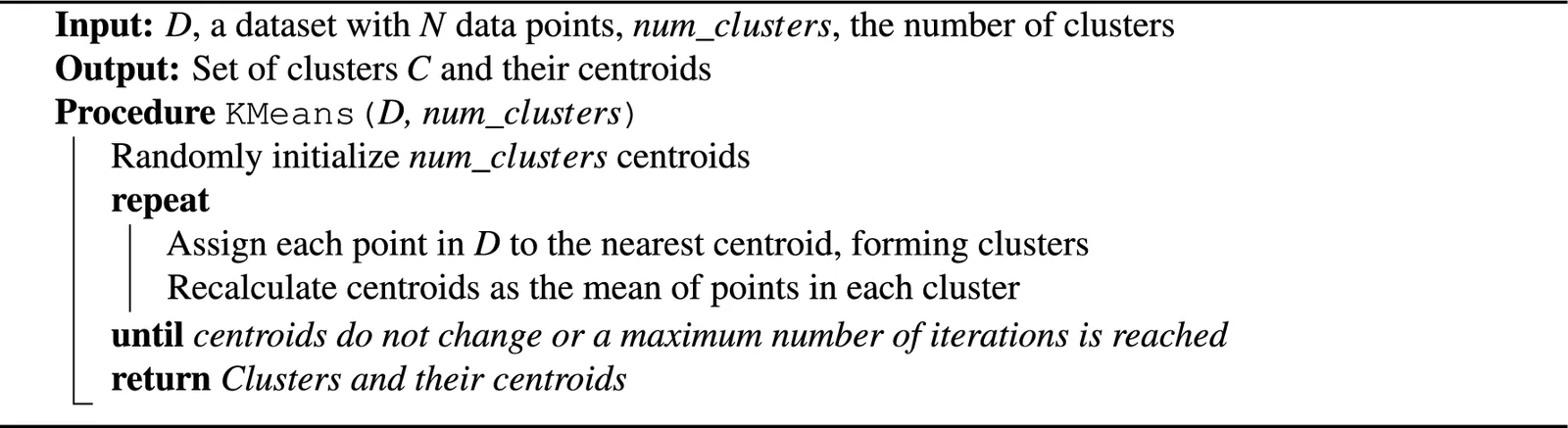
K-Means

**Algorithm 3:**
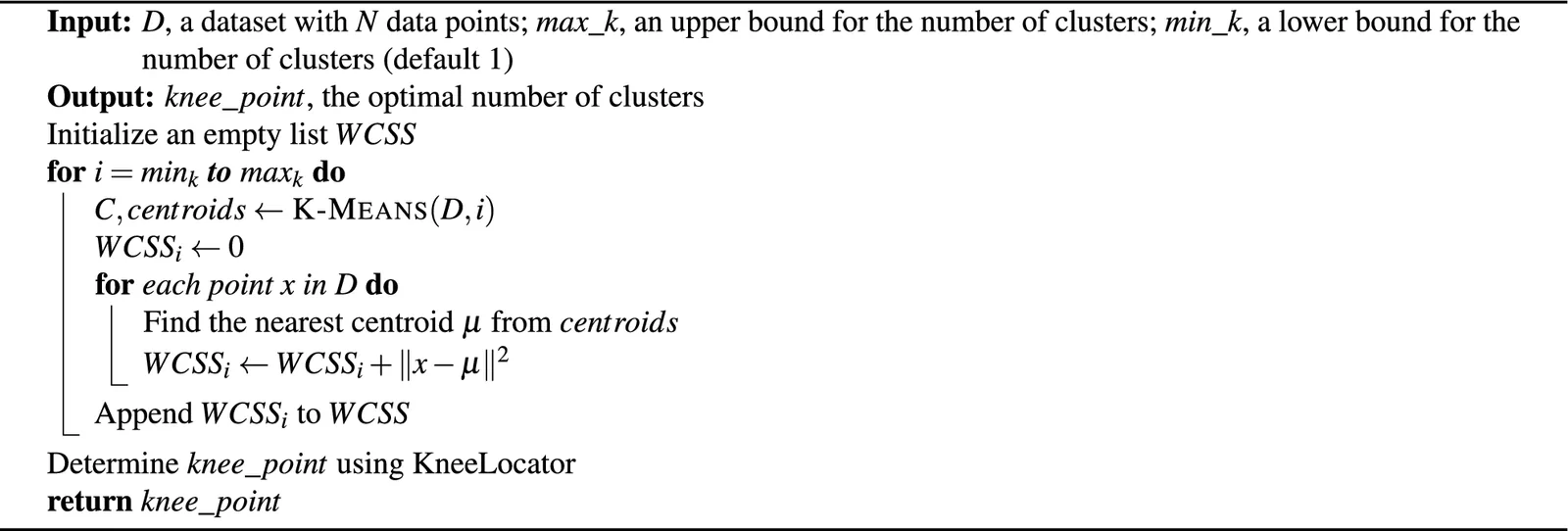
Elbow method to find optimal clusters

### Data labelling and WDM classification with TTT-SSR

In this phase of the framework, we present an automatic WDM classification approach.

#### Data labelling

One objective of the optimised K-means clustering technique is to support automatic class labelling of unseen WDMs. During inference, let $x_u$ denote an unseen WDM, and let $f_{\theta }$ be the trained encoder that projects $x_u$ into the latent space, where nearest-centroid label assignment is performed.

The latent representation of $x_u$ is given by

7 $$\begin{aligned} z_u = f_\theta (x_u), \end{aligned}$$

where $z_u \in \mathbb {R}^d$. Let $\{\mu _k\}_{k=1}^{K}$ denote the centroids of the *K* clusters in the latent space, and let $\Vert \cdot \Vert _2$ denote the Euclidean norm. The nearest centroid is identified as

8 $$\begin{aligned} k^{*} = \arg \min _{k} \left\| z_u - \mu _k \right\| _2, \end{aligned}$$

where $k^{*} \in \{1,\dots ,K\}$. The predicted class label is then obtained as

9 $$\begin{aligned} \hat{y}_u = \sigma \!\left( k^{*}\right) , \end{aligned}$$

where $\sigma (\cdot )$ denotes the cluster-to-class mapping. The resulting $\hat{y}_u$ serves as an automatic pseudo-label for the unseen WDM.

#### Test-Time Training Semi-Supervised Refinement (TTT-SSR) for classification performance improvement

Although the optimised clustering analysis captures the underlying structure of wafer defect classes effectively, it may not always provide sufficiently reliable labelling for practical WDM classification, particularly when data distributions are complex and defect patterns evolve. To address this limitation, we employ TTT-SSR as a semi-supervised refinement strategy during the test phase. The main idea is to combine a limited labelled training subset with pseudo-labelled unseen samples so that the classifier can learn more reliable decision boundaries beyond nearest-centroid assignment alone. The labelled training subset is defined as

10 $$\begin{aligned} \mathscr {D}_{\text {train}}^{\text {cl}} = \{\, (z_i, y_i) : (x_i, y_i) \in \mathscr {D}_{\ell } \,\}, \end{aligned}$$

where $\mathscr {D}_{\ell }$ is the limited labelled subset, $z_i = f_{\theta }(x_i)$, and $y_i$ is the ground-truth class label of $x_i$. Since the pseudo-label $\hat{y}_u$ for each unseen sample has already been obtained through nearest-centroid assignment and cluster-to-class mapping in the Section “Data labelling”, the pseudo-labelled unseen set is written as

11 $$\begin{aligned} \mathscr {D}_{\text {test}}^{\text {pseudo}} = \{\, (z_u, \hat{y}_u) : x_u \in \mathscr {D}_{\text {test}}^{u} \,\}. \end{aligned}$$

Although the labelled subset and unseen subset contain different samples, both are represented in the same latent feature space through the encoder and in the same class-label space. These two sets are then combined to refine a discriminative classifier at test time. In this work, we employ an XGBoost classifier^31^, denoted $h_\phi$, which is trained on

12 $$\begin{aligned} \mathscr {D}_{\text {TTT\_SSR}} = \mathscr {D}_{\text {train}}^{\text {cl}} \;\cup \; \mathscr {D}_{\text {test}}^{\text {pseudo}}. \end{aligned}$$

XGBoost optimisation implicitly minimises a regularised objective of the form

13 $$\begin{aligned} \mathscr {L}(\phi ) = \sum _{(z,y) \in \mathscr {D}_{\text {TTT\_SSR}}} \ell \!\left( h_\phi (z),\, y \right) \;+\; \Omega (\phi ), \end{aligned}$$

where $\ell (\cdot ,\cdot )$ is the logistic loss used by XGBoost and $\Omega (\phi )$ is the model’s tree-based regularisation term. Rather than correcting pseudo-labels individually, the XGBoost classifier uses both the limited labelled training subset and the pseudo-labelled unseen samples to refine decision boundaries in the latent space. This helps reduce part of the noise introduced by nearest-centroid assignment and improves WDM classification during the test phase^31^.

### Performance evaluation

To assess the effectiveness of the proposed methodology, we employ appropriate evaluation metrics for both the clustering and classification.

#### Clustering performance evaluation metrics

Clustering quality is evaluated using standard metrics that assess label consistency and structural coherence: homogeneity, completeness, V-measure, adjusted mutual information (AMI), and silhouette score^32^.

- **Homogeneity** (*h*): Measures if each cluster contains only one class.
  14 $$\begin{aligned} h = 1 - \frac{H(C|K)}{H(C)} \end{aligned}$$
  where *C* is the set of true labels, *K* is the set of predicted clusters, and *H(· )* denotes entropy.
- **Completeness** (*c*): Measures if all members of a class are in the same cluster.
  15 $$\begin{aligned} c = 1 - \frac{H(K|C)}{H(K)} \end{aligned}$$
- **V-measure** (*v*): Harmonic mean of homogeneity and completeness.
  16 $$\begin{aligned} v = 2 \cdot \frac{h \cdot c}{h + c} \end{aligned}$$
- **Adjusted Mutual Information (AMI)**: Adjusts mutual information for chance.
  17 $$\begin{aligned} \text {AMI} = \frac{\text {MI} - E(\text {MI})}{\max (H(C), H(K)) - E(\text {MI})} \end{aligned}$$
  where $\text {MI}$ is mutual information between *C* and *K*, and $E(\text {MI})$ is its expected value under random labeling.
- **Silhouette Score** (*s*): Evaluates how well samples fit within their clusters versus others.
  18 $$\begin{aligned} s = \frac{b - a}{\max (a, b)} \end{aligned}$$
  where *a* is the mean intra-cluster distance, and *b* is the mean nearest-cluster distance.

#### Classification performance evaluation metrics

For classification performance, we use well-known evaluation metrics including accuracy, precision, recall, and F1-score, which are defined as follows^16^:

19 $$\begin{aligned} \text {Accuracy}&= \frac{\text {TP} + \text {TN}}{\text {TP} + \text {TN} + \text {FP} + \text {FN}},\end{aligned}$$

20 $$\begin{aligned} \text {Precision}&= \frac{\text {TP}}{\text {TP} + \text {FP}},\end{aligned}$$

21 $$\begin{aligned} \text {Recall}&= \frac{\text {TP}}{\text {TP} + \text {FN}},\end{aligned}$$

22 $$\begin{aligned} \text {F1-Score}&= \frac{2 \times \text {Precision} \times \text {Recall}}{\text {Precision} + \text {Recall}}, \end{aligned}$$

where $\text {TP}$, $\text {TN}$, $\text {FP}$, and $\text {FN}$ denote true positives, true negatives, false positives, and false negatives, respectively.

## Results and discussion

In this section, we present a detailed evaluation of the proposed clustering and classification framework on a publicly available benchmark WDMs dataset.

### Dataset description

The proposed approach is evaluated on the MixedWM38 benchmark dataset, which comprises 38015 WDMs spanning a diverse set of defect classes commonly observed in semiconductor manufacturing^16^. These defect classes cover a variety of patterns, including multiple single defects and mixed defect clusters, which signal different manufacturing anomalies. Each sample contains 2704 features (52*×*52), yielding 38,015 samples in total. Thus, the dataset dimensions are 2704*×*38015 after reshaping each input pattern, which is considered significantly high-dimensional. This high dimensionality requires an efficient feature extraction method, such as the proposed autoencoder-based feature learning. To ensure robust evaluation, 67% of the dataset is randomly selected as the training set and the remaining 33% is designated as the test set. For TTT-SSR, a limitted number of samples from training partition (i.e., 10% in this study) is randomly selected as the labelled subset while the remaining samples are treated as unlabelled data for representation learning and clustering. This partitioning is repeated independently for each experiment.

Wafer maps are preprocessed by normalising pixel values to a range of 0 to 1 and reshaping the images into flattened vectors for compatibility with the deep learning models. Although the dataset provides labels for each wafer map, they are used only for performance evaluation and validation, not during the unsupervised learning process.

### Clustering analysis performance

**Fig. 2:**
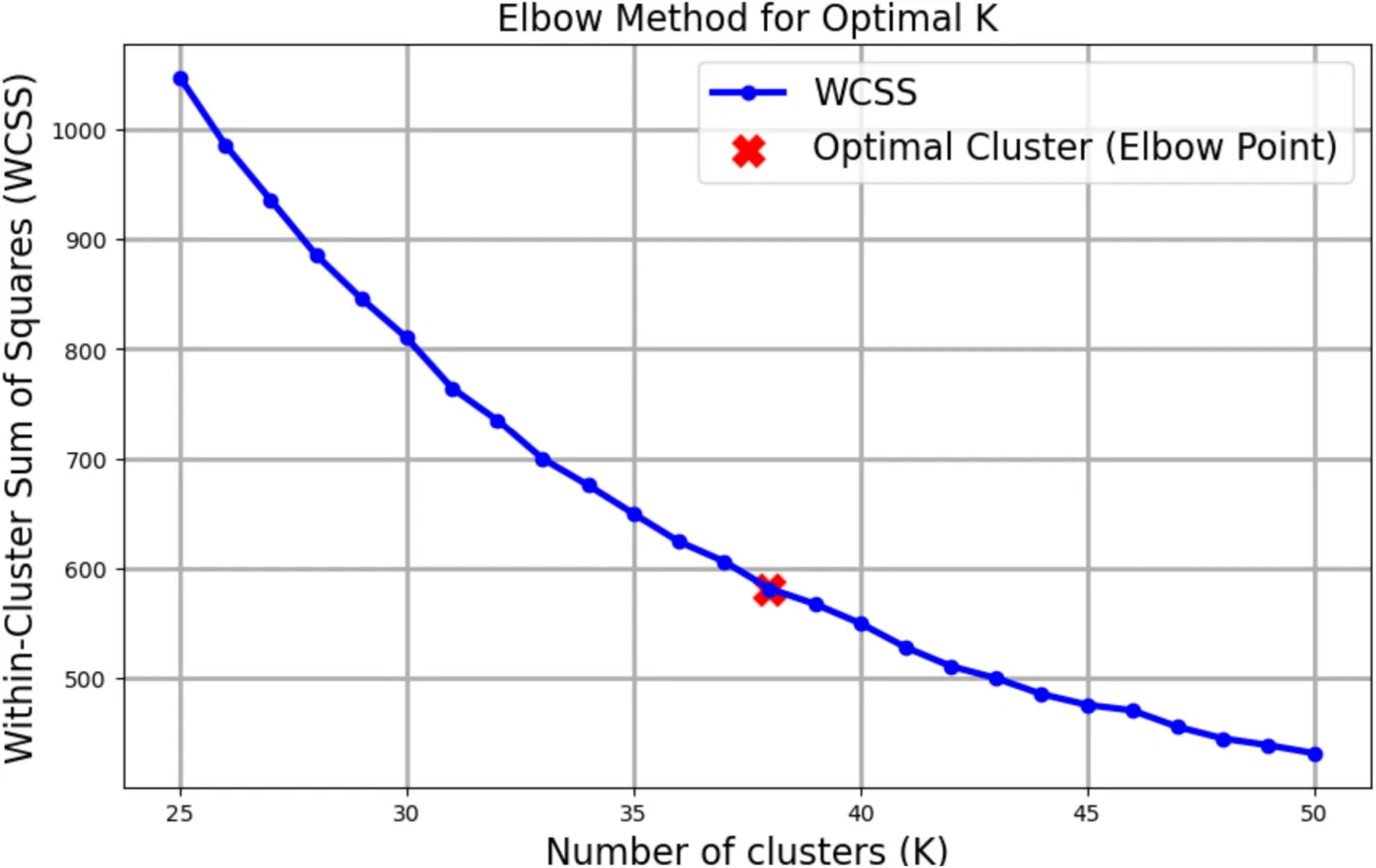
Results of the Elbow-based optimised K-means clustering algorithm: WCSS versus *K* curve used to determine the optimal number of clusters.

Figure 2 presents results of the Elbow-based clustering analysis, where the within-cluster sum of squares decreases as the number of clusters increases and shows a visible change in slope around *K = 38*. Since the MixedWM38 benchmark covers the most frequently occurring defect patterns and their combinations, comprising 38 categories, this value appears to provide a reasonable trade-off between cluster compactness and model complexity for this dataset. However, the correspondence between the selected cluster number and the number of benchmark classes are viewed as an empirical observation for this dataset rather than as a general indication of exact class recovery.

Clustering quality was further evaluated through a numerical comparison of different feature extraction approaches. Table 3 summarises clustering performance using the metrics defined in Eqs. (11)–(15). As shown in Table 3, the proposed method achieved the highest values across the main clustering metrics, with homogeneity of 0.754, completeness of 0.815, V-measure of 0.783, and AMI of 0.782. Across these metrics, the proposed method showed average improvements of 81.05% over the All Features + K-means baseline and 72.45% over the PCA + K-means baseline. Although silhouette scores were comparable across methods, the consistently higher homogeneity, completeness, V-measure, and AMI values indicate that the proposed latent representation provides stronger structural separation of wafer defect patterns and improved clustering quality overall.

**Table 3:** Comparison of clustering performance across different feature extraction methods using the metrics defined in Equations (14)–(18).

Method	*h*	*c*	*v*	AMI	*s*	Average
All Features + K-Means^33^	0.363	0.409	0.385	0.380	0.470	0.401
PCA Features + K-Means^34^	0.386	0.423	0.404	0.399	0.495	0.421
Autoencoder Features + Optimised K-Means (Proposed)	**0.754**	**0.815**	**0.783**	**0.782**	0.495	**0.726**

**Fig. 3:**
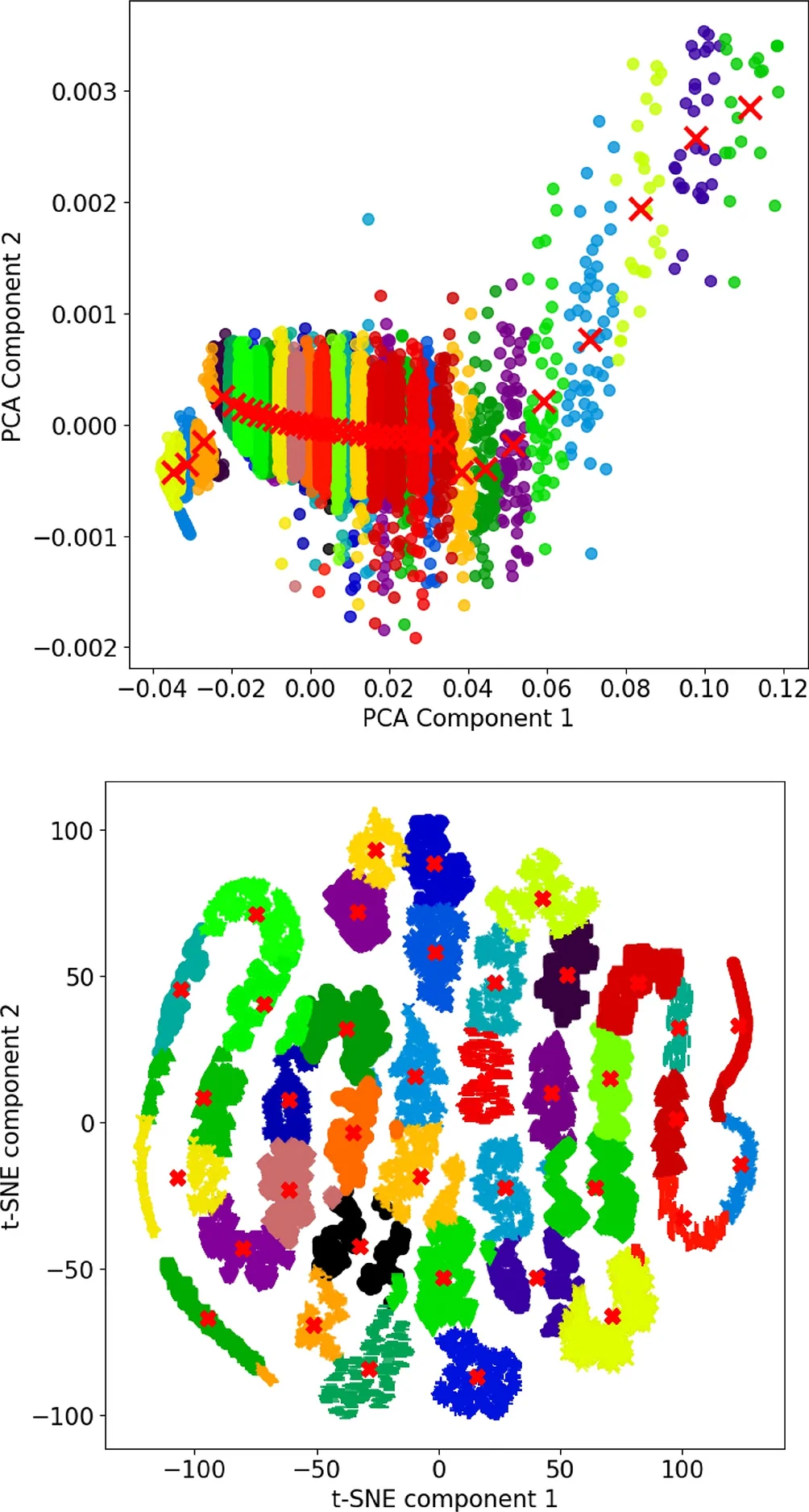
Comparison of PCA (top) and proposed autoencoder-based feature extraction (bottom) for K-means clustering of semiconductor wafer defects.

In addition to the numerical evaluation, we assess clustering performance using 2-D visualisations in Figure 3. The PCA-based representation (top) shows considerable overlap between clusters, indicating limited ability to separate complex defect patterns. In contrast, the autoencoder-based feature space (bottom) produces more compact and well-separated clusters, with centroids (marked by ‘x’) located away from overlapping regions. These visual observations support the quantitative results, where the autoencoder-based method achieves higher homogeneity and completeness.

### Classification results

Another main objective of this study is to improve the classification accuracy of the proposed automatic WDM recognition framework during the testing phase. Table 4 reports the performance of the proposed model across fault classes, selected by the proposed automatic clustering method, in terms of precision, recall, and F1-score. The results demonstrate that the model achieves consistently high classification performance, with most fault classes exhibiting F1-scores above 0.93.Notably, several classes, including Faults 0, 4, 5, 7, 8, and 20, achieved near perfect F1 scores of 0.98, highlighting the model’s ability to accurately distinguish dominant fault patterns.

Table 5 compares the proposed framework with three representative approaches: the self-supervised contrastive learning model SWaCo^6^, the deformable convolutional network DC-Net^16^, and AL-UDR^24^. SWaCo uses self-supervised contrastive pre-training on large collections of unlabelled wafer bin maps and then fine-tunes a classifier on a small labelled subset, whereas DC-Net is a fully supervised deformable CNN designed for mixed-type wafer defect patterns. AL-UDR combines a supervised classifier for known patterns, DBSCAN-based clustering for unknown patterns, and classifier updating through active learning. As shown in Table 5, the proposed framework achieves the strongest overall performance, with an accuracy of 0.95, precision of 0.95, recall of 0.95, and an F1-score of 0.94. By comparison, SWaCo attains an accuracy of 0.92, precision of 0.92, recall of 0.90, and an F1-score of 0.89, whereas DC-Net achieves an accuracy of 0.93, precision of 0.94, recall of 0.95, and an F1-score of 0.93. The AL-UDR baseline reports an accuracy of 0.94, precision of 0.91, recall of 0.91, and an F1-score of 0.91. Overall, these results indicate that the proposed framework provides the most accurate and best-balanced WDM classification among the selected self-supervised, supervised, and active-learning-based models.

**Fig. 4:**
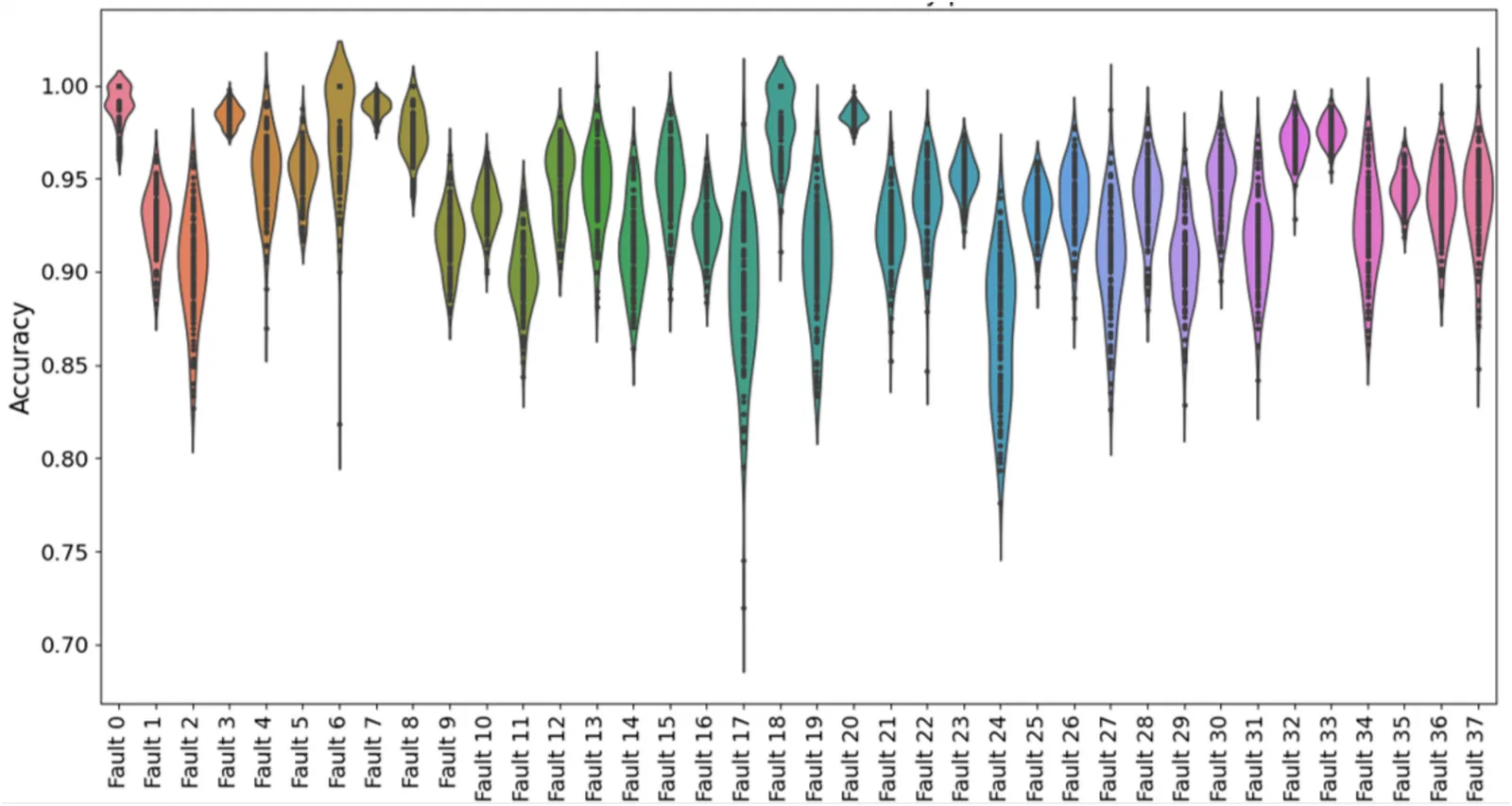
Distribution of classification accuracy for 38 fault classes over 100 bootstrap samples. Each violin depicts the density and spread of accuracy.

Figure 4 shows the distribution of class-wise classification accuracy for the all fault classes over 100 bootstrap samples using violin plots. Each violin represents the density and spread of accuracy values for a given fault class. Overall, the results demonstrate strong and stable class-wise performance, with most accuracy distributions concentrated above 0.90. The compact distributions observed for many classes indicate reliable predictions under resampling and support the robustness of the proposed framework. Although few classes, including Fault 17, Fault 24, and Fault 27, exhibit relatively wider spreads or lower tails, their performance remains generally high. Therefore, Figure 4 provides additional evidence that the proposed framework achieves not only high overall classification accuracy, but also robust performance across the majority of defect categories.

**Table 4:** Fault class-wise performance of the proposed model based on precision, recall, F1-score, and accuracy.

**Fault Class**	**Precision**	**Recall**	**F1-score**
Fault 0	0.98	0.98	0.98
Fault 1	0.97	0.93	0.95
Fault 2	0.92	0.94	0.93
Fault 3	0.96	0.98	0.97
Fault 4	0.98	0.98	0.98
Fault 5	0.97	0.98	0.98
Fault 6	0.94	1.00	0.97
Fault 7	0.98	0.98	0.98
Fault 8	0.98	0.99	0.98
Fault 9	0.94	0.91	0.93
Fault 10	0.95	0.92	0.94
Fault 11	0.93	0.94	0.93
Fault 12	0.95	0.96	0.95
Fault 13	0.92	0.98	0.95
Fault 14	0.93	0.97	0.95
Fault 15	0.93	0.93	0.93
Fault 16	0.87	0.90	0.88
Fault 17	0.95	0.95	0.95
Fault 18	0.96	0.95	0.96
Fault 19	1.00	0.96	0.98
Fault 20	0.98	0.98	0.98
Fault 21	0.96	0.95	0.96
Fault 22	0.91	0.96	0.93
Fault 23	0.93	0.96	0.94
Fault 24	0.92	0.91	0.91
Fault 25	0.93	0.94	0.94
Fault 26	0.95	0.89	0.92
Fault 27	0.93	0.88	0.90
Fault 28	0.96	0.92	0.94
Fault 29	0.91	0.94	0.93
Fault 30	0.95	0.93	0.94
Fault 31	0.88	0.90	0.89
Fault 32	0.96	0.93	0.95
Fault 33	0.97	0.96	0.96
Fault 34	0.92	0.93	0.92
Fault 35	0.93	0.92	0.92
Fault 36	0.94	0.92	0.93
Fault 37	0.95	0.95	0.95
**Accuracy**	0.95		
**Weighted Avg.**	0.95	0.94	0.94

**Table 5:** Classification performance comparison with state-of-the-art models based on Accuracy, Precision, Recall, and F1 Score.

**Model**	**Accuracy**	**Precision**	**Recall**	**F1 Score**
Self-Supervised^6^	0.92	0.92	0.90	0.89
DC-Net^16^	0.93	0.94	0.95	0.93
AL-UDR^24^	0.94	0.91	0.91	0.91
Proposed	0.95	0.95	0.95	0.94

### Overall discussion

The proposed method demonstrates strong performance in both clustering and classification. This performance is mainly associated with the combined effect of unsupervised feature learning, optimised clustering, and test-time refinement. In the clustering phase, the use of autoencoder-based features significantly enhances the separability of fault patterns compared to traditional PCA- or raw feature-based methods. The Elbow analysis shows a visible change in slope around *K = 38*, which appears to provide a reasonable balance between cluster compactness and model complexity on the MixedWM38 benchmark. However, the agreement between the selected cluster number and the number of benchmark classes is interpreted as an empirical observation. Visual inspection further supports these findings, as the autoencoder-based feature space yields more compact and better separated defect groupings.

One objective of the optimised clustering stage is to provide an initial basis for automatic labelling through nearest-centroid assignment. However, the reliability of these initial labels may decline when unseen samples exhibit complex distributions or evolving defect patterns. In this context, TTT-SSR serves as a refinement step by combining the limited labelled subset with pseudo-labelled unseen samples, which helps improve classification performance. Class-wise evaluation shows that most F1-scores exceed 0.93, with several fault categories achieving near-perfect values. The violin plot illustrates stable accuracy distributions with low variance across bootstrap samples. When compared with the selected benchmark methods, including SWaCo, DC-Net and AL-UDR, the proposed framework shows competitive overall performance, with the highest accuracy and F1-score in this evaluation. Overall, these findings suggest that the framework offers a practical approach to automatic WDM recognition. The current dataset covers the most frequently occurring defect patterns and their combinations, comprising 38 categories. This offers a strong and practically meaningful basis for evaluating the proposed framework under representative wafer defect conditions. At the same time, broader validation under real industrial drift, changing operating conditions, and genuinely new defect categories remains an important direction for future work.

## Conclusion

This study presented an automatic semiconductor wafer defect recognition framework for complex manufacturing environments. In the training phase, a deep autoencoder is used to automatically extract features from predominantly unlabelled wafer defect maps, and an Elbow-guided optimised K-means method is used to determine the number of clusters automatically. This allows the framework to learn the underlying defect structure from unlabelled data and to provide an initial basis for automatic labelling. In the testing phase, unseen WDMs are first labelled through nearest-centroid assignment in the latent space. In practical applications, however, these initial labels may become less reliable under complex and evolving conditions. To address this, they are further refined using XGBoost-based test-time semi-supervised refinement. Results on the MixedWM38 benchmark show improved clustering quality and a classification accuracy of 95%, with competitive performance across the main evaluation metrics. Future work will explore a fully unsupervised WDM recognition pipeline with reduced dependence on labelled data. This will include density-based clustering, improved latent-space representations, and adaptive test-time refinement to enhance robustness against class imbalance and overlapping defect patterns.

## Data Availability

The datasets used in this study are available in the Kaggle repository, https://tinyurl.com/MixedWDM.
